# Biocompatibility and magnetic resonance imaging characteristics of carbon nanotube yarn neural electrodes in a rat model

**DOI:** 10.1186/s12938-015-0113-6

**Published:** 2015-12-21

**Authors:** Yi Guo, Wanru Duan, Chao Ma, Changqing Jiang, Yikuan Xie, Hongwei Hao, Renzhi Wang, Luming Li

**Affiliations:** Department of Neurosurgery, Peking Union Medical College Hospital, Beijing, China; Institute of Basic Medical Sciences, Chinese Academy of Medical Sciences, School of Basic Medicine, Peking Union Medical College, Beijing, China; Department of Neurosurgery, Xuan Wu Hospital affiliated to Capital Medical University, Beijing, China; Institute of Man–Machine and Environmental Engineering, School of Aerospace, Tsinghua University, Beijing, China

**Keywords:** Carbon nanotubes, Electrochemistry, Deep brain, Stimulation/instrumentation, MRI, Electrode, Neuron depletion, Inflammatory response

## Abstract

**Background:**

Implantation of deep brain stimulation (DBS) electrodes is a landmark therapy for movement disorders and some mental conditions. Compared to conventional platinum–iridium (Pt–Ir) electrodes, carbon nanotube yarns (CNTY) electrodes have improved stability and interface characteristics with less distortion during high field strength MRI. Sprague–Dawley rat models were used to examine the*in vivo* histological and imaging properties of biocompatible CNTY throughout the subacute period.

**Methods:**

Sprague–Dawley rats received CNTY (*n* = 16) or Pt–Ir control (*n* = 16) electrodes. Behavioral markers, body weight, and survival were recorded. Comparative histology (HE, NeuN, CD68, and GFAP) was performed at 1, 6, and 12 weeks post-implantation; 3.0T MRI was performed at 1 and 12 weeks.

**Results:**

Of 32 rats, 30 (15 per group) survived implantation without reduced activity, paralysis, or incapacity to feed. Following implantation, progressive decreases in macrophage activation and neuron-depleted margins surrounding electrodes were observed in both groups. Inflammatory marker expression (CD68) was significantly lower in rats with implanted CNTY electrodes compared to controls at all time points. CNTY electrodes also caused less inflammation and shallower depths of macrophage penetration and neural disruption relative to the interface. Artifacts and distortion were observed on MRI of Pt–Ir but not CNTY electrodes.

**Conclusions:**

CNTY electrodes exhibited reduced inflammatory margins compared to Pt–Ir electrodes throughout the subacute period, indicating reduced initial trauma, better overall biocompatibility, and reduced fibrous tissue formation. Coupled with less MRI distortion, CNTY electrodes may be useful alternatives when there is a need to monitor electrode placement by MRI.

**Electronic supplementary material:**

The online version of this article (doi:10.1186/s12938-015-0113-6) contains supplementary material, which is available to authorized users.

## Background

Platinum–iridium (Pt–Ir) alloy, gold, and stainless steel electrodes are the most commonly used implantable neural electrode for deep brain stimulation (DBS), a landmark therapeutic approach for neuromodulation of diverse movement disorders and mental health conditions including Parkinson’s disease (PD), dystonia, and epilepsy [1]. In 1986, DBS of the motor thalamus and ventral intermedius nucleus (VIM) was initially used as a means of treating refractory tremor in PD [2], though it has since been used in the internal globuspallidus (GPi) and subthalamic nucleus (STN) as well as other basal ganglia nuclei [3].

The material selection of electrodes is critical to the electrochemical properties of the interface between neural tissues and implanted electrodes. While conventional Pt–Ir alloys exhibit good biocompatibility and resistance to corrosion, use of platinum reduces current, and iridium oxide electrodes may disintegrate or shed during long-term implantation [4]. A number of metallic, diamond-like, ceramic, and polymer coatings have been proposed, which have discreet advantages and disadvantages [5]. In addition, some metals may have toxic tissue effects [6].

The electrode–electrolyte interfaces can be modeled as simple circuits of serial capacitance and serial resistance along with Faradic resistance [7]. The electrode–electrolyte interface is clinically important because its characteristics play a role in both safety and efficacy of DBS treatments by influencing recovery, stability, electrical stimulation radius, and activation density in vivo [8]. These procedures have, however, been limited by current density hot spots at the electrode surface, short-term drift, aging, and corrosion at the interface, which can adversely affect the delivery of consistent charge and increase inflammatory processes adjacent to the electrode [9].

Stainless steel electrodes, and to a lesser extent Pt–Ir electrodes, are associated with significant image distortion and artifacts on magnetic resonance imaging (MRI) [10], limiting clinicians’ ability to verify placement and stability of implanted electrodes. Thus, there is a need for advanced materials that have superior biocompatibility and that are compatible with high field strength MRI (3.0T and higher). Contacts along the plane and tip of DBS electrodes can be used for chronic stimulation and typically are able to be separately activated to ensure stimulation at the correct location, enabling minor modification even after implantation. MRI and stereotactic X-ray are, however, crucial intra- and post-operative indicators of electrode position [11].

Carbon nanotube yarns (CNTY) electrodes are non-metallic and initial MRI results have shown that the CNTY electrodes show very few artifacts, with as much as a 62 and 74 % reduction in artifacts on spin echo (SE) and gradient echo (GE) sequences [12]. CNTYs have recently been proposed as an alternative to stainless and Pt–Ir electrodes, since they display superior capacitance at the interface due to their uniform porous structure and good stability under continuous electrical stimulation, as well as compatibility with MRI [13]. Coupled with good biocompatibility [14], these results suggest that CNTY may be a promising solution to overcome the limitations of conventional electrodes for DBS.

From an engineering perspective, a number of common materials found in electrodes are based on the manufacturing of thin films and are incompatible with commercial fabrication processes, which make these devices prohibitively expensive to produce in a meaningful scale and may thus limit their practical utility in biomedicine [5]. Recent improvements of production strategies involving mechanical twisting of lightweight carbon nanotubes (CNTs) to generate uniform CNTYs with good electrical conductivity, high mechanical strength, and flexibility have made the manufacturing of CNTY electrodes more feasible on a large scale [15]. There are clear structural and electronic characteristics of carbon nanotubes that can improve sensitivity, selectivity, spatial resolution, and electron transfer kinetics compared to both conventional metal alloy and carbon-fiber microelectrodes typically used for in vivo DBS procedures [16]. This may indicate that the interface of properly processed CNTY electrodes have superior and more stable properties for in vivo use, as well as generating little to no distortion on MRI. In medical applications, invasive diagnosis instruments are typically single-use and are thus required to be inexpensive to manufacture as large batches, a capability that may be possible for contemporary CNTYs unlike their predecessors.

In this study, the CNTYs were prepared by the twisting and shrinking method described by Liu et al. [17] from super-aligned CNT arrays synthesized using the chemical vapor deposition (CVD) method. To obtain a hydrophilic surface, CNTYs were soaked into boiling 65 % HNO for 3 min. Both the untreated and treated CNTYs were then cut into 40 cm segments, and the electrodes were made by wrapping these yarns tightly around a polyurethane spindle to achieve the same shape as the Pt–Ir electrodes. The electrochemical properties of the electrodes were not tested in this study since it has been tested previously [13]. Since the materials and methods used were identical and prepared in the same laboratory, the properties were assumed to be identical.

While information about the characteristics of these materials are abundant in the recent literature, very little research has been done to characterize the progressive changes at the interface of CNTYs compared to conventional Pt–Ir metal electrodes. Therefore, this study was conducted to better understand the progressive histological and electrochemical characteristics of the tissue-electrode interface over the subacute period after implementation of CNTY electrodes in live rat brains. Both histological characteristics and distortion on MRI images were examined.

## Methods

### Experimental design

Sprague–Dawley (SD) rats aged 2–6 months were randomly assigned to the CNTY (*n* = 16) or control Pt–Ir (*n* = 16) groups. Animals were treated with synthesized CNTY or commercial Pt–Ir alloy electrodes (Medtronic DBS electrodes Models 3387 and 3389, Medtronic Inc., Minneapolis, MN, USA), respectively. Electrodes were implanted into the STN of each animal. Animals were housed separately and allowed ad libitum access to food and water after a recovery period of 2 h, and were studied throughout the subacute period (12 weeks). This study was conducted in accordance with the requirements for animal care and handling of the Peking Union Medical College Hospital Committee for Animal Research and the Institutional Ethics Committee.

### CNTY synthesis and electromechanical assessments

CNTY were synthesized by mechanical twisting and shrinking in solvent using the method proposed by Liu et al. [17] with high quality CNTs provided by the Chinese Academy of Sciences, resulting in CNTY with good mechanical strength (up to 1 GPa), uniform cross-sectional circle (50 μm in diameter), excellent conductivity, and high flexibility. Electrode was made by wrapping CNTY around polyurethane tube (Estane^®^ 58238 TPU, Lubrizol, Ohio, USA) with a diameter of 1.3 mm. The electrode has comparable mechanical characteristics as medtronic 3389 (Additional file 1: Figure S1). Electrochemical impedance spectrum (EIS) was measured to be 95 and 71 Ω for the untreated and acid treated CNTY at 1 kHz.

### Passive electrode implantation

Each animal was anesthetized by intraperitoneal injection of 1 % sodium pentobarbital (40 mg/kg), and its head was fixed by a conventional three-dimensional fixation in a stereotaxic instrument. The median skin was incised to expose the anterior fontanelle. According to Paxinos & Watson coordinates [18], the STN position was determined to be 3.8 mm behind the anterior fontanelle, 2.5 mm to the midline, and 6 mm subdural. Passive CNTY or Pt–Ir electrodes were implanted slowly and fixed at the set position.

### Behavioral and health assessments

Overall survival and post-electrode placement behavioral patterns indicative of central nervous system damage (reduced activity, paralysis, or incapacity to feed) were recorded for all animals immediately after passive electrode implantation and at 12 weeks. The Neurological severity scores (NSS) was used to assess eventual neurological injuries [19]. Weight gain was assessed at 1, 6, and 12 weeks for all animals as an indicator of general health.

### Histological and immunohistochemical testing

For tissue sampling, experimental animals were anesthetized by intraperitoneal injection of 1 % sodium pentobarbital (40 mg/kg). Brain tissue specimens were sampled at 1, 6, and 12 weeks from the STN part that was directly adjacent to the electrodes by tissue biopsy. Briefly, anesthetized rats underwent rapid aortic root perfusion of about 150 mL phosphate buffer until outflow from the right atrial appendage getting clear. Then the perfusion continued with 200 mL 4 % formaldehyde solution. Then the brain tissue was surgically removed and fixed in 4 % formaldehyde for 12–24 h. Histological samples were prepared as cryosections and sliced into X-mm sections for hematoxylin and eosin (HE) staining. Specimens were also labeled for CD68 for macrophage density (#MCA341R, 1:250, AbDSerotec, Kidlington, UK), glial fibrillary acidic protein (GFAP) for astrocyte proliferation (#AB1540, 1:1000, Chemicon, Temecula, CA, USA), and NeuN for neuron survival (#ZM0352, 1:100, Zhongshanjinqiao, Beijing, China).

Following anesthesia at 12 weeks, the animals were euthanized using 4 % paraformaldehyde and perfused with PBS. Brain was excised in whole and fixed with 4 % paraformaldehyde for 12–24 h. STN tissues containing electrodes were excised, and routinely processed for paraffin embedding. Consecutive sections of 6 μm were prepared and attached to poly-l-lysine-coated slides. After dewaxing with xylene, sections were stained with HE. The other sections were incubated with antibodies against CD68, GFAP, or NeuN at 4 °C overnight, followed by addition of an appropriate dilution of horseradish peroxidase-conjugated secondary antibody followed by 3, 3′-diaminobenzidine (DAB). Slides were mounted, observed by light microscopy (BX51, Olympus, Japan) at 100×.

### MRI

MRI scanning was performed at 1 and 12 weeks on living animals using a Philips MRI Systems 72 3.0 TX (Amsterdam, The Netherlands). Prior to MRI scanning, rats were anesthetized with intraperitoneal injection of 1 % sodium pentobarbital (40 mg/kg), and the animal’s head was fixed in a coil with the body axis at the centerline. T1- and T2-weighted coronal and sagittal scans were performed with slices thickness of 2.0 mm and interval space of 0.2 mm. Parameters of T1-weighted imaging were: TE = 20 ms, TR minimum = 500 ms, TR maximum = 700 ms, ACQ matrix M × P = 216 × 120. Parameters of T2-weighted imaging were: TE = 110 ms, TR = 2000 ms, ACQ matrix M × P = 216 × 175. Location and occurrence of artifacts, noise, and general quality of T1- and T2-weighted images were recorded.

### Statistical analysis

Data were processed using SPSS 11.0 (SPSS Inc., Chicago, IL, USA) and were presented as mean ± standard error of mean (SEM). Differences were analyzed with one-way analysis of variance (ANOVA) and Student’s *t*-tests. *P*-values less than 0.05 were considered statistically significant.

## Results

### General condition of animal subjects

Of the 32 rats that underwent electrode implantation, two (6.3 %) rats (one in the CNTY group and one in the control group) did not survive due to acute blockage of the respiratory tract during the procedure. All other rats survived to 12 weeks. No significant differences were observed between the CNTY and control rats forage (4.3 ± 0.36 vs. 4.4 ± 0.41 months), body weight (242 ± 22 vs. 245 ± 25 g), and survival rate (93.8 %). No significant differences in mechanical strength, uniformity, cross-sectional dimensions (50 μm in diameter), conductivity, or flexibility were observed in the synthesized CMTY electrodes.

### Behavioral and health assessments

**Fig. 1:**
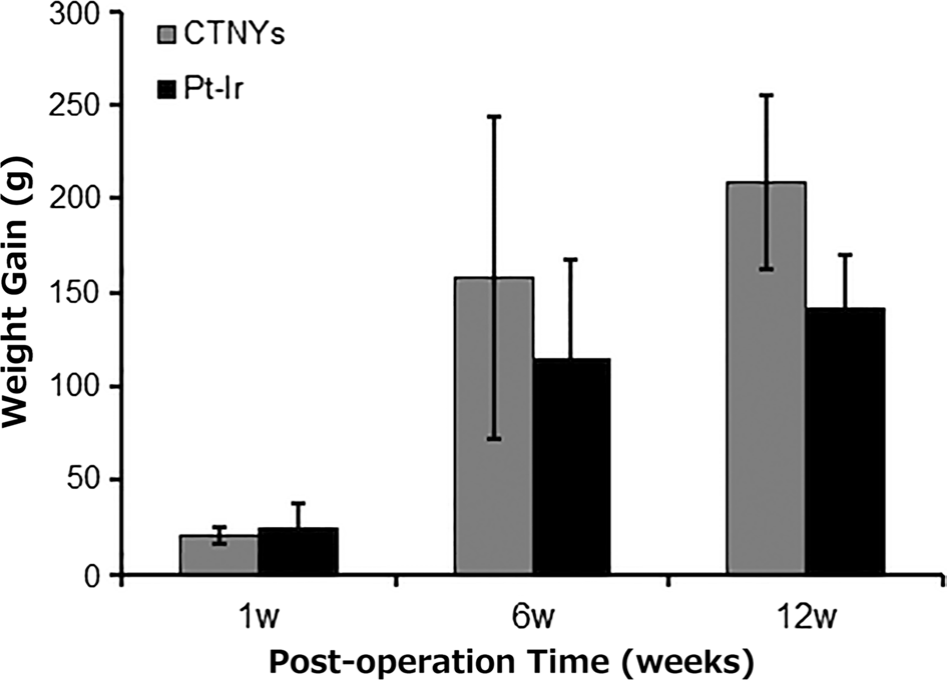
Post-operative weight gain following electrode implantation at 1, 6, and 12 weeks

Of the 15 surviving rats in each group, 10 CNTY and 9 controls exhibited reduced eating and drinking for around 3 days when allowed free access to food and water after surgery. No animals in either group exhibited slow movement, tremor, skew, rotation, change in aggression, or other behaviors indicative of central nervous system damage. The NSS was 0 for all surviving animals. While weight gain was not significantly different between the CNTY and control groups at any time point, animals in the CNTY group exhibited slightly higher weight gain at 6 and 12 weeks (Fig. 1). These findings indicate that there is likely no significant effect on the general health between the two groups during the subacute period, though larger groups may potentially indicate some benefits of biocompatible electrodes in the acute period after implantation.

### Gross histological observations

**Fig. 2:**
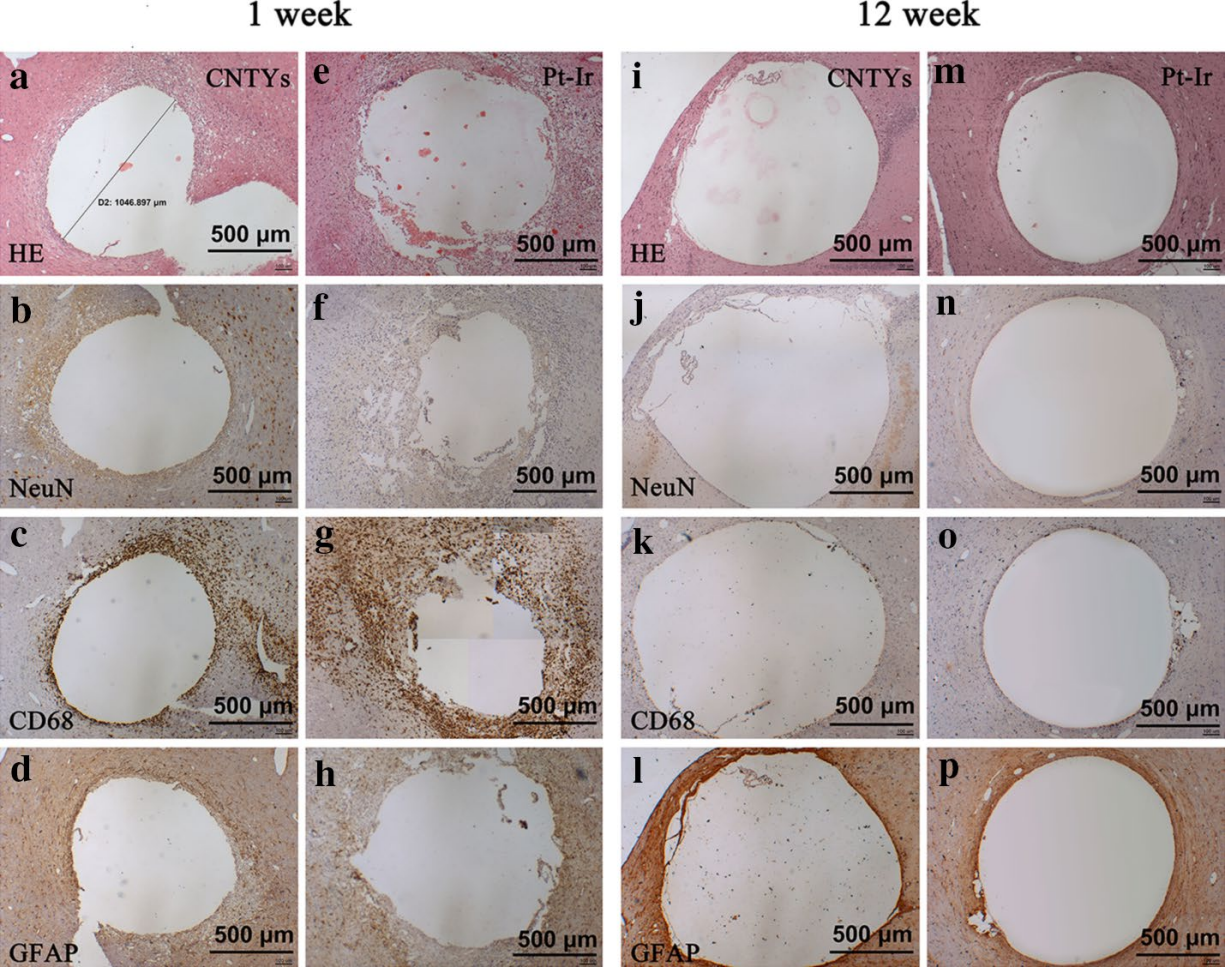
Gross histological tissues results surrounding implanted CNTY and control Pt–Ir electrodes at week 1 and 12 (×100). **a** HE staining of tissues around CNTY electrode at 1 week; **b** NeuNimmunostaining of tissues around CNTY electrode at 1 week; **c** CD68 immunostaining of tissues around CNTY electrode at 1 week; **d** GFAP immunostaining of tissues around CNTY electrode at 1 week; **e** HE staining of tissues around Pt–Ir electrode at 1 week; **f** NeuNimmunostaining of tissues around Pt–Ir electrode at 1 week; **g** CD68 immunostaining of tissues around Pt–Ir electrode at 1 week; **h** GFAP immunostaining of tissues around Pt–Ir electrode at 1 week; **i** HE staining of tissues around CNTY electrode at 12 weeks; **j** NeuNimmunostaining of tissues around CNTY electrode at 12 weeks; **k** CD68 immunostaining of tissues around CNTY electrode at 12 weeks; **l** GFAP immunostaining of tissues around CNTY electrode at 12 weeks; **m** HE staining of tissues around Pt–Ir electrode at 12 weeks; **n** NeuNimmunostaining of tissues around Pt–Ir electrode at 12 weeks; **o** CD68 immunostaining of tissues around Pt–Ir electrode at 12 weeks; **p** GFAP immunostaining of tissues around Pt–Ir electrode at 12 weeks

Histological slides were examined under light microscopy (HE, NeuN, CD68, and GFAP staining) in the CNTY and control (Pt–Ir) groups. These tests consistently revealed gross differences between groups at 1 and 12 weeks (Fig. 2). Most notably, the inflammatory cell layers adjacent to the control electrodes were significantly thicker at 1 week on HE staining, indicative of inflammation and acute tissue injury (Fig. 2a, e). By 12 weeks, the margins were more similar, indicative of reduced tissue inflammation in both groups; however, non-neuronal areas remained discrepant on HE staining (Fig. 2i, m). At 1 and 12 weeks, NeuN staining consistently revealed a notably thinner and more disperse set of viable neurons (brown) around the CNTY electrodes (Fig. 2b, j) compared to Pt–Ir electrodes (Fig. 2f, n). Correspondingly, the CD68 macrophage density (brown) surrounding control electrodes was notably larger and denser that that found around CNTY electrodes (Fig. 2c, k), confirming an elevated acute inflammatory response intensity in the control group (Fig. 2g, o). Similar astrocyte proliferation, indicative of chronic inflammatory reaction, was observed by GFAP staining in both groups (Fig. 2d, h, l, p). Overall, histological results indicated slightly reduced inflammatory processes in the acute (1 week) period using CNTY electrodes, though electrodes in both groups exhibited reduced inflammation by 12 weeks. Evidences of chronic cytotoxic effects were not observed in either group.

### Change in neuron-depleted tissue area

**Fig. 3:**
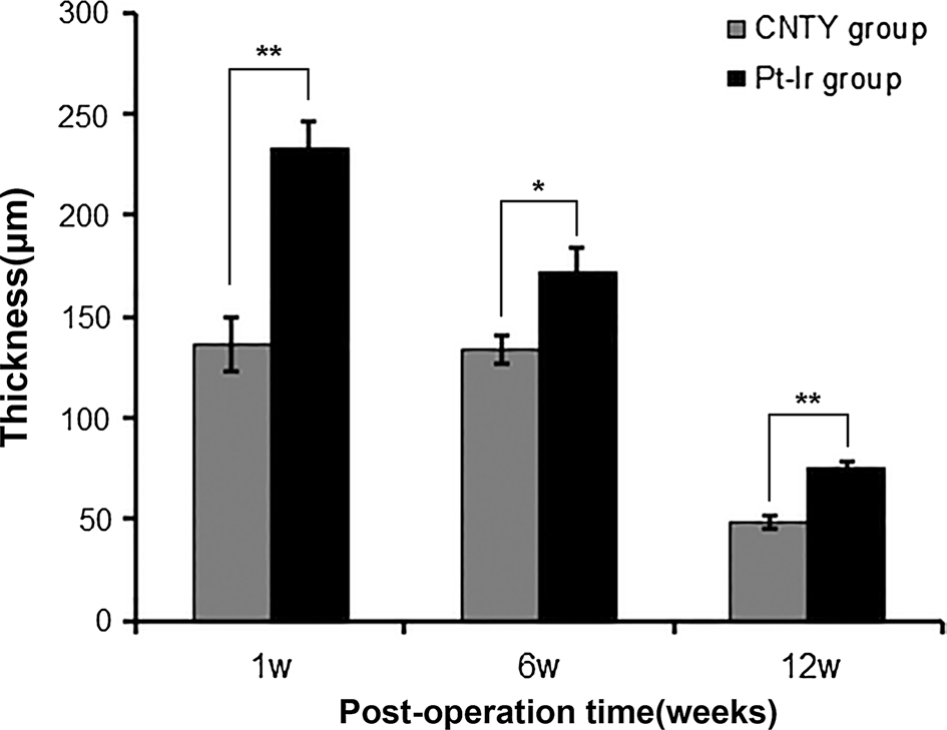
Thickness of neuron-depleted areassurrounding CNTY and control Pt–Ir electrodes. At 1, 6, and 12 weeks after electrode implantation, the thickness of the neuron-depleted layer of brain tissue surrounding the electrodes were compared. * *P* < 0.05, ** *P* < 0.01

Neuron-depleted tissue area adjacent to both CNTY and control electrodes (neuron-depleted regions are centrally composed of protein deposition, glial cells, and macrophages formed due to the trauma of electrode placement) progressively decreased in both groups from 1 to 12 weeks. Notably, animals treated with CNTY exhibited significantly lower non-neuronal tissue area at all time points (*P* < 0.05; Fig. 3), and had greater diffusion of viable neurons than controls, apparent on gross histology, at all time points. GFAP-positive astrocytes around CNTY electrodes were in the chronic phase and showed comparatively reduced proliferation.

### Macrophage activation

**Fig. 4:**
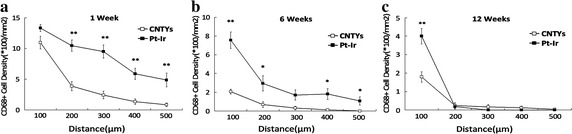
Macrophage activation (CD68-positive density) at 1, 6, and 12 weeks around CNTY and control Pt–Ir electrodes. **a** CD68-positive celldensity 1 week after implantation. **P < 0.01. **b** CD68-positive celldensity 6 weeks after implantation. *P < 0.05; **P < 0.01. **c** CD68-positive celldensity 12 weeks after implantation. **P < 0.01

CD68-positive cell distribution (macrophage activation) decreased as a function of the distance from the electrode in both groups, with consistently lower macrophage densities with the CNTY compared tocontrol electrodes (Fig. 4a, b, c). While initial 1- and 6-week results indicated significantly higher penetration of macrophage activation, 12-week results were similar in both groups.

### MRI

While minimal artifacts and noise were seen on MRI of CNTY electrodes, Pt–Ir alloy electrodes produced characteristically larger artifacts (approximately 3 mm in diameter around the electrode, obscuring the tissue details immediately surrounding the electrode) and greater diffuse noise on MRI for both T1- and T2-weighted images.

## Discussion

**Fig. 5:**
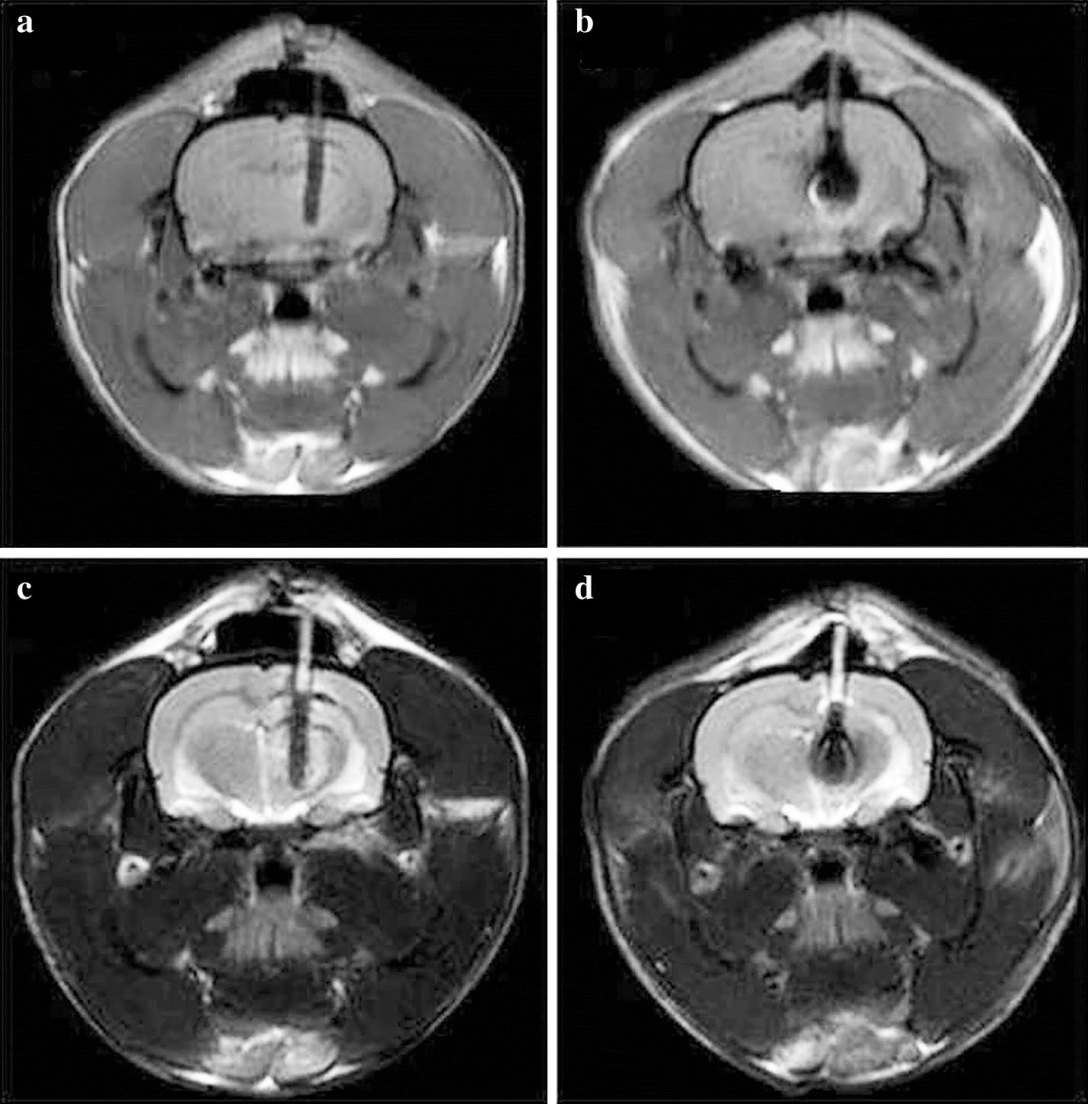
MRI comparison of Pt–Ir and CNTY electrodes at 1 week after implantation. **a**, **c** T1- and T2-weighted images of CNTY electrodes, respectively; **b**, **d** T1- and T2-weighted images of control Pt–Ir alloy electrodes, respectively

**Fig. 6:**
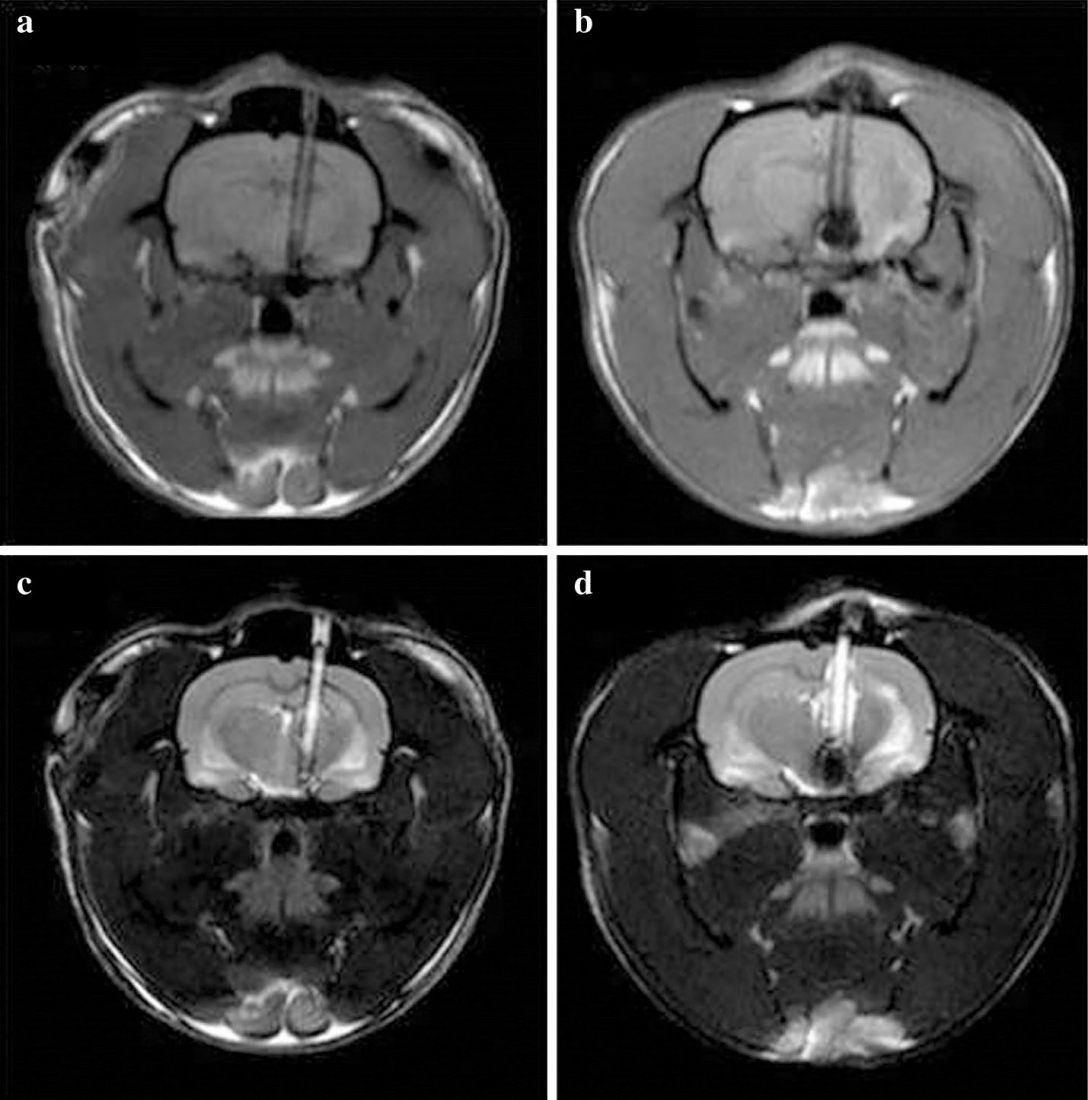
MRI comparison of Pt–Ir and CNTY electrodes at 12 weeks after implantation. **a**, **c** T1- and T2-weighted images of CNTY electrodes, respectively. **b**, **d** T1- and T2-weighted images of control Pt-Ir alloy electrodes, respectively

Rats implanted with CNTY or Pt–Ir alloy electrodes evidenced similar survival rates and progressive recovery after implantation. Rats with CNTY electrodes, however, showed comparatively lower levels of inflammation on histological examinations and narrower bands of neural cell depletion adjacent to the electrodes after 12 weeks. At 1 and 12 weeks, CNTY electrodes produced minimal MRI artifacts, allowing for clear visualization of the surrounding tissues and confirmation of electrode placement. This study provides preliminary evidence that the use of CNTY electrodes may result in limited inflammation, reduced localized neural depletion, and superior MRI visualization, making it relatively easier to ensure the correct position of the electrodes and minimize placement trauma (Figs. 5, 6).

DBS electrodes can be constructed of many different kinds of metals such as noble metals like gold and platinum (designed to be minimally toxic) as well as stainless steel. Varied materials are commercially available and described in the literature, in which novel materials and manufacturing methods have been proposed to yield specific electrochemical characteristics suitable for the use of neural electrodes in vivo. These include surface characteristics (a rough surface can reduce interface impedance and provide superior electrical potential stability in electrodes) and conductive properties. Though conductive polymers and even CNT coatings can achieve improved electrochemical properties over conventional metal alloy electrodes, these remain impaired by issues of coating detachment and immune response, particularly for long-termuse [4, 5].

### CNTY electrode biocompatibility

Neural electrode biocompatibility is characterized by limited inflammatory response, high interface surface area with porous electrode surface, and minimized neuron depletion around the electrode [5]. While biocompatibility of CNT is still controversial, recent studies have suggested that highly oriented 50-nm-thick CNT sheets and yarns produced with minimal residual content of catalytic transition materials have superior biocompatibility and do not induce toxic tissue effects like many metals [6]. Furthermore, these materials have been demonstrated to support long-term cell growth in a variety of cell types including skin fibroblasts, Schwann cells, postnatal cortical neurons [20], and cerebellar neurons [6]. Properly designed CNTY electrodes could not only prohibit excessive inflammation but also possibly induce neuronal growth, at similar levels as those observed using materials like polyornithine-coated glass [6].

In this study, HE and IHC findings are supported by findings of previous studies, which reported that CNT materials have potential for both in vitro and in vivo applications [6, 21]. Galvan-Garcia et al. [6] reported that CNTs can be used as a contact surface for the growth of many cells. Schwann cells and dorsal root ganglion cells grow and migrate on the surface of the CNTs [6]. The results of this study demonstrate that after implantation in animal brain tissue, the neuron-depleted areas surrounding CNTY electrodeswere narrower and the inflammatory reaction was milder compared to the area surrounding Pt–Ir alloy electrodes. These findings support the biocompatibility of CNTY electrodes.

### Acute response to bioelectrode placement

The mechanisms of the acute phase response, or virtually instantaneous reactions of the body triggered by implanted electrodes, are well characterized [22]. This initial injury results in highly variable degrees of tissue trauma such as damage and rupture of blood vessels, localized swelling, and even localized neural death. Dramatically increased serum amyloid A and C-reactive protein during this period can occur as a result of inflammatory reaction to the interface materials [23]. Subsequent vasodilation and congestion can cause prolonged secretion of immunoglobulins, complements, and antithrombin from the cells adjacent to the electrode, leading to inflammation and increasing the size of the adverse tissue effect margin around the newly placed electrode [24]. Neutrophils, monocytes, and lymphocytes gradually migrate and are recruited around the foreign body, and activated macrophages gather on the electrode surface [25].

In this study, a large number of inflammatory cells around the electrode were clearly visible by routine HE staining at 1 week, and CD68 staining also showed macrophage activation around both the CNTY and Pt–Ir electrodes, though the density and depth of aggregation varied between electrode types. These results suggest that the acute phase response triggered by CNTY is weaker than that which is elicited by the conventional Pt–Ir electrode. These findings provide preliminary evidence that CNTY electrodes could have the potential to reduce the postoperative inflammatory responses such as fever and leukocytosis. Further trials, however, are necessary to verify the biocompatibility of CNTY electrodes in humans, and under different electric current conditions (rather than as passively used in this study).

### Subacute progression following bioelectrode placement

While initial placement of electrodes is critical, the subsequent formation of multinucleated foreign-body giant cells (FBGCs) by large numbers of macrophages unsuccessful in engulfing a foreign body can promote fibroblast proliferationand collagen and proteoglycan synthesis [26]. Fibrous cysts may form to isolate the foreign body, resulting in progressive changes in electrical resistivity based on gradual shift in character of the electrode-tissue interface, increasing the amount of current required to elicit the expected effects [27]. Therefore, the degree of fibrosisis an important factor that affects implanted electrode performance. Thus, this study examined the progression from 1 to 12 weeks, demonstrating gross histological changes in the thickness of the fibrous layers around the electrodes (neuron-depleted region) that were notably smaller when CNTY electrodes were used compared to Pt–Ir alloy electrodes.

### MRI of CNTY electrodes

Due to the difference in magnetic susceptibility of metal-containing electrodes, large MRI artifacts are observed around the electrodes, which affect the recognition of electrode position and identification of the surrounding structures [5]. This initial evidence suggests that CNTYs may have beneficial characteristics compared to Pt–Ir and other noble metal alloys. Noble metals can produce characteristic distortion and artifacts in induced magnetic fields, which become more apparent on MRI using high static field strengths of 3.0T and above and may vary according to position/orientation in reference to each other and to the plane of the applied magnetic field [28].

Susceptibility differences have been identified as the dominant cause of distortion due to artifacts on MRI of neural electrodes [12]. Because CNTY electrodes are made of non-metallic carbon nanomaterials, their magnetic susceptibility is similar to model solutions. In addition, the spinning method used to generate CNTYs reduces the effect of the magnetic field on the electrode, so that MRI artifact signals are extremely weak, as demonstrated by in vitro studies [12]. In this study, MRI scanning showed that implanted CNTY electrodes produced virtually no MRI artifacts, enabling clear visualization of pathological changes around the electrodes. In contrast, artifacts from the Pt–Ir electrodes distorted the image around the electrodes, making it difficult to determine the exact localization of the electrode position with accuracy in vivo.

The small sample size and anatomical difference in animal models require that these findings be further tested under clinical conditions. In addition, a better control group would have been achieved using commercially available carbon-fibermicro-electrodes, but no electrode of the appropriate size was available for rats. Additional studies will have to be performed in large animal models to perform an adequate comparison of these electrodes. The present study was designed only to assess the biocompatibility of CMTY and no stimulation current was applied; this will be examined in future studies. Finally, histological changes were performed at 1 and 12 weeks only since repeated tissue biopsy could further injure the animals and bias the results. Nevertheless, these preliminary results strongly suggest that CNTY may provide superior performance in terms of biocompatibility and margins of neural depletion and macrophage activation compared to conventional Pt–Ir electrodes.

## Conclusion

This study demonstrates that histological changes including inflammation and neural depletion are progressive processes over time in the subacute period from 1 to 12 weeks after implantation of brain electrodes. CNTY electrodes resulted in initial and final inflammation levels and neural depletion margins that were significantly smaller than those produces by comparable conventional Pt–Ir electrodes. CNTY is known to be strong, flexible, and have good electrochemical properties, with similar capacitance and stability as Pt–Ir electrodes [13]. CNTY electrodes also produced few visible MRI artifacts compared to Pt–Ir electrodes under in vivo conditions.

## Additional file

10.1186/s12938-015-0113-6 CNTY electrode compared to Pt-I electrode.

## Abbreviations

Pt–Ir: Platinum–iridium
DBS: deep brain stimulation
PD: Parkinson’s disease
MRI: magnetic resonance imaging
VIM: ventral intermedius nucleus
GPi: globuspallidus
STN: subthalamic nucleus
SE: spin echo
GE: gradient echo
CNTYs: carbon nanotube yarns
