# Supplementary figures and images for: Biocompatibility and magnetic resonance imaging characteristics of carbon nanotube yarn neural electrodes in a rat model
Source: Biomed Eng Online. 2015 Dec 21;14:118. doi: 10.1186/s12938-015-0113-6 (PMC4687330; doi:10.1186/s12938-015-0113-6)

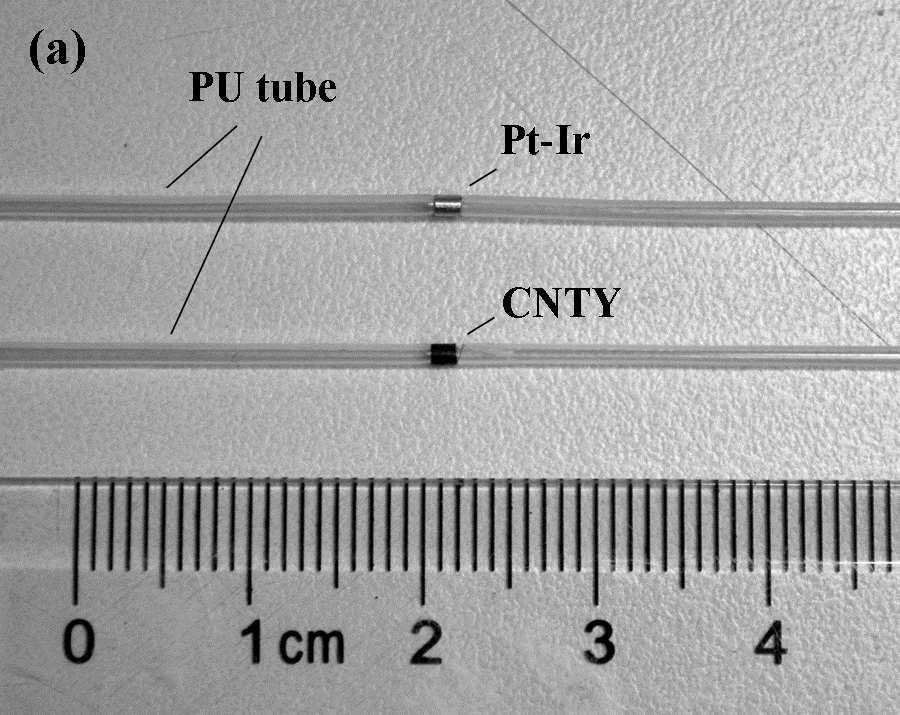


**Supplemental figure S1** CNTY electrode compared to Pt-Ir electrode.

Supplement: Supplementary file 1 — 10.1186/s12938-015-0113-6 CNTY electrode compared to Pt-I electrode. [file 12938_2015_113_MOESM1_ESM.docx]
